# Fracture resistance and failure mode of three esthetic CAD-CAM post and core restorations

**DOI:** 10.1186/s12903-024-04273-y

**Published:** 2024-05-03

**Authors:** Islam T. Fathey, Amir Shoukry Azer, Islam M. Abdelraheem

**Affiliations:** Division of Fixed Prosthodontics, Conservative Dentistry Department, Faculty of Dentistry, Alexandria University, P. O. Box: 21527, Champollion St., Azarita, Alexandria Egypt

**Keywords:** Post and core, Cad-cam, Glass fiber-reinforced composite, Polymer-infiltrated ceramic-network, Polyetheretherketone

## Abstract

**Background:**

The rising demand for improved aesthetics has driven the utilization of recently introduced aesthetic materials for creating custom post and core restorations. However, information regarding the fracture resistance of these materials remains unclear, which limits their practical use as custom post and core restorations in clinical applications.

**Aim of the study:**

This study aimed to evaluate the fracture resistance of three non-metallic esthetic post and core restorations and their modes of failure.

**Materials and methods:**

Thirty-nine single-rooted human maxillary central incisors were endodontically treated. A standardized post space preparation of 9mm length was performed to all teeth to receive custom-made post and core restorations. The prepared teeth were randomly allocated to receive a post and core restoration made of one of the following materials (*n*=13): glass fiber-reinforced composite (FRC), polyetheretherketone (PEEK) and polymer-infiltrated ceramic-network (PICN). An intraoral scanner was used to scan all teeth including the post spaces. Computer-aided design and computer-aided manufacturing (CAD-CAM) was used to fabricate post and core restorations. Post and core restorations were cemented using self-adhesive resin cement. All specimens were subjected to fracture resistance testing using a universal testing machine. Failure mode analysis was assessed using a stereomicroscope and SEM. The data was statistically analyzed using One-Way ANOVA test followed by multiple pairwise comparisons using Bonferroni adjusted significance level.

**Results:**

Custom PEEK post and core restorations displayed the least fracture load values at 286.16 ± 67.09 N. In contrast, FRC exhibited the highest average fracture load at 452.60 ± 105.90 N, closely followed by PICN at 426.76 ± 77.99 N. In terms of failure modes, 46.2% of specimens with PICN were deemed non-restorable, while for PEEK and FRC, these percentages were 58.8% and 61.5%, respectively.

**Conclusions:**

Within the limitation of this study, both FRC and PICN demonstrated good performance regarding fracture resistance, surpassing that of PEEK.

## Background

Endodontically treated teeth (ETT) exhibit an elevated susceptibility to fracture, primarily attributable to the substantial loss of tooth structure resulting from decay and cavity preparation. Furthermore, the occurrence of biochemical and structural alterations within dental structure can impede the long-term viability of ETT [1]. In order to mitigate the risk of fracture in ETT, restorative approaches involving cuspal coverage, such as onlays, overlays, endocrowns, and single crown restorations, are recommended [2]. However, in instances where there is insufficient dental structure to support a restoration, the utilization of post and core restorations becomes necessary to provide the requisite retention for a single crown restoration [3].

While prefabricated posts are a commonly employed solution for enhancing the retention of core materials within ETT, they face a notable challenge due to their circular cross-sectional shape, which does not conform precisely to the elliptical nature of root canals [4]. This incongruence results in an increased thickness of the cement layer between the post and root dentin, ultimately diminishing post retention and clinical efficacy [5]. On the other hand, custom post and core restorations handle the issue of excessive cement space and offer superior retention and internal fit [6]. Nevertheless, the intricate fabrication processes associated with custom posts may introduce distortions and irregularities to the metal surface following casting, potentially compromising the adaptation of the restoration [7].

Computer-Aided Design and Computer-Aided Manufacturing (CAD-CAM) technology has emerged as an effective method for fabricating post and core restorations with exceptional precision [8, 9]. This technology facilitates the utilization of various CAD-CAM materials, including zirconia, glass ceramic, and hybrid ceramics. It is worth noting that while zirconia and glass ceramic are among the materials that can be employed, they exhibit high moduli of elasticity, which can increase the risk of root fracture [10, 11].

Glass fiber-reinforced composite (FRC), polyetheretherketone (PEEK) and polymer-infiltrated ceramic-network (PICN) have emerged as promising alternatives to traditional materials like metal, zirconia, and glass ceramics in the field of restorative dentistry [12]. One notable advantage of these materials is that their modulus of elasticity closely approximates that of dentin, allowing them to function as effective shock absorbers, thereby reducing the stresses transmitted to the underlying tooth structure [13, 14]. The evolution of these materials, coupled with the integration of digital impressions and CAD-CAM systems, has made them a viable and practical option for restoring ETT [15–17].

Unlike prefabricated glass fiber posts containing unidirectional glass fibers combined within epoxy resin, the CAD-CAM FRC blank comprises a mixture of multidirectional glass fibers embedded within an epoxy resin matrix, exhibiting a modulus of elasticity of 26 GPa [18]. Polyetheretherketone (PEEK), possessing a modulus of elasticity of 4 GPa, [19, 20] represents a thermoplastic, partially crystalline high-performance polymer (HPP) [21]. It comprises an aromatic benzene molecular chain linked by interconnected ether and ketone functional groups [22]. Hybrid ceramic refers to a polymer-infiltrated ceramic network (PICN) exhibiting a modulus of elasticity of 30 GPa. The primary fine structure comprises a ceramic network constituting 86% by weight, reinforced by an acrylate polymer network. Both networks are intricately interlinked, forming a fully integrated composite [23, 24].

The field of digital dentistry has advanced with the aim of enhancing precision and expediting the production process [25]. Traditionally, the use of CAD-CAM technology for fabricating customized posts was constrained to the scanning of plaster models derived from conventional impressions [26]. However, alternative approaches have been proposed by different researchers, including the use of a traditional silicone impression that is scanned to manufacture a customized CAD-CAM post and core [27]. In contrast, the in vitro study conducted in this context employed an intraoral scanner for the direct scanning of the post space, thereby achieving a fully digital workflow [9].

The primary objective of this in vitro study was to assess the fracture resistance and failure patterns exhibited by three non-metallic esthetic custom post and core restorations, all of which have been produced using CAD-CAM technology. The null hypothesis posited that no difference would be observed in fracture resistance among these three materials.

## Materials and methods

The materials used in the current study are listed in (Table 1). Power analysis was performed using a statistical software program (GPower 3.1.9.4; Henrich Heine University Dus-seldorf) [28]. Sample size was estimated assuming alpha error= 5% and study power= 80%. Eid et al. [29] reported a mean fracture resistance of post and core material manufactured from fiber reinforced composite of 367.06 ± 72.34, while AlKhatri et al. [30] reported a mean fracture resistance of 271.06 ± 69.57 in polymer infiltrated ceramic network group. Teixeira et al. [20] reported mean ± SD fracture resistance= 379.4 ± 119.8 in case of PEEK post and core. Calculations were performed using the formula stated below [31]. Based on comparison of means, with an effect size of 0.55, the sample size was calculated to be 12 per group, increased to 13 to make up for laboratory processing errors. The total sample size required = number of groups × number per group= 3 x 13 = 39 [32].

**Table 1:** Materials used

**Trade** **Name**	**Manufacturer**	**Composition**	**Lot No.**
**Trilor** **(FRC)**	Trilor; Bioloren	Epoxy resin matrix (25% vol), multi directional glass fiber reinforcement (75% vol)	1919
**breCAM. BioHPP** **(PEEK)**	Bredent	80% PEEK with 20% nanoceramic filler	511807
**Vita Enamic** **(PICN)**	Vita Zahnfabrik	Polymer-infiltratedfeldspatic ceramicnetwork material (UDMA, TEGDMA) with 86 wt% ceramic	43260 44690 49490
**TOTALCEM** **(Resin cement)**	Itena	UDMA, Bis-GMA, TEGDMA, 4-methacryloxyethyltrimellitic acid, barium glass, fumed silica	4283-42HQBSETR
**SILAN-IT** **(Silane coupling agent)**	Itena	of 3-methacryloxypropyl-trimethoxysilane 5 wt.%, and water\ethanol solution containing acetic acid of PH 4, 95 wt. %	4203-09PFXS
**CERAM-ETCH** **(Hydrofluoric acid)**	Itena	9% buffered hydrofluoric acid	4203-08PFXE
**Visio.Link** **(visiolink primer)**	Bredent	MMA, PETIA, dimethacrylates, photoinitiators (diphenyl(2,4,6,-trimethylbenzoyl)phosphinoxide)	192004

$$N={\left(\frac{\left({t}_{1- ^{\alpha }\!\left/ \! _{2}\right.}+{t}_{1-\beta }\right)\sigma }{d}\right)}^{2}$$

N is the required sample size, t is the two-tailed T distribution, $\sigma$ represents the standard deviation, $\alpha$ is the given probability value, $\beta$ represents the study power, and d is the effect size.

This study received ethical approval from the Institutional Review Board of the Faculty of Dentistry at Alexandria University (IORG: 0008839, approval no. 0439-05/2022). Sample preparation and examination was done at the Conservative Dentistry Department laboratory at the Faculty of Dentistry, Alexandria University. A total of thirty-nine human maxillary central incisors, which had been extracted due to periodontal reasons, were obtained from the Department of Oral Surgery at the Faculty of Dentistry, Alexandria University. The selected teeth underwent a comprehensive visual and radiographic assessment to verify their absence of prior endodontic treatment, restorations, carious lesions, structural cracks, or signs of internal resorption. Additionally, this examination served to confirm the presence of a straight, single root canal with a fully matured apex. Cracks in teeth were identified visually through the utilization of a 6x magnifying loupe, coupled with transillumination employing an LED light source directed perpendicular to the tooth [33]. This method facilitated the detection of cracks by observing the diffraction of light when it intersected the crack, thereby enabling the precise localization of the crack and the exclusion of affected specimens [34]. Following these assessments, the teeth were subjected to a thorough ultrasonic cleaning procedure.

### Specimens preparation

The collected specimens were preserved in a 0.9% saline solution at room temperature to prevent desiccation. Subsequently, the teeth were carefully sectioned, using a double-sided diamond disk (Dynex Separating discs Brillant; Renfert), approximately 2 mm coronal to the cement-enamel junction. This cutting process was carried out with a low-speed handpiece, while ensuring a constant flow of copious coolant. Each tooth underwent endodontic treatment utilizing rotary files (PROTAPER-GOLD; Dentsply Sirona) up to size F5. Canal irrigation was performed using 5.25% sodium hypochlorite. Following this, paper points were used to dry the canals, and obturation was carried out using the lateral compaction technique with gutta-percha cones (Spident; Meta Biomed Co) and resin sealer (AD Seal; Meta Biomed) [9]. The remaining structure of every tooth was prepared with a 2 mm ferrule coronal to the CEJ. To remove the gutta-percha, Gates Glidden drill size 3 (Dentsply Sirona) was used. Subsequently, post spaces were standardized at a length of 9 mm with a black color-coded drill (Unimetric; Dentsply Sirona). The teeth were embedded in an auto-polymerizing acrylic resin (Acrostone Acrylic Material-Cold Cure; ACROSTONE Co) to a level 2mm apical to the CEJ to simulate the bone level.

### Grouping of the specimens

All 39 teeth were randomly allocated to receive post and core restorations using one of the following materials (*n*=13): FRC (Trilor; Bioloren), PEEK (breCam.BioHPP; Bredent), and PICN (Vita Enamic; VITA Zahnfabrik). The randomization procedures were performed by a statistical website (Randomizer.org).

### Scanning of the post space

The post spaces that had been prepared were scanned using an intraoral scanner (MEDIT i700; Medit), to acquire standard tessellation language (STL) files [8, 9]. The justification for utilizing intraoral scanners for post space scanning stems from their ability to precisely capture intricate impressions of the post space inside the tooth, as stated in previous studies [8, 35, 36].

### Designing and milling of post and core restorations

**Fig. 1:**
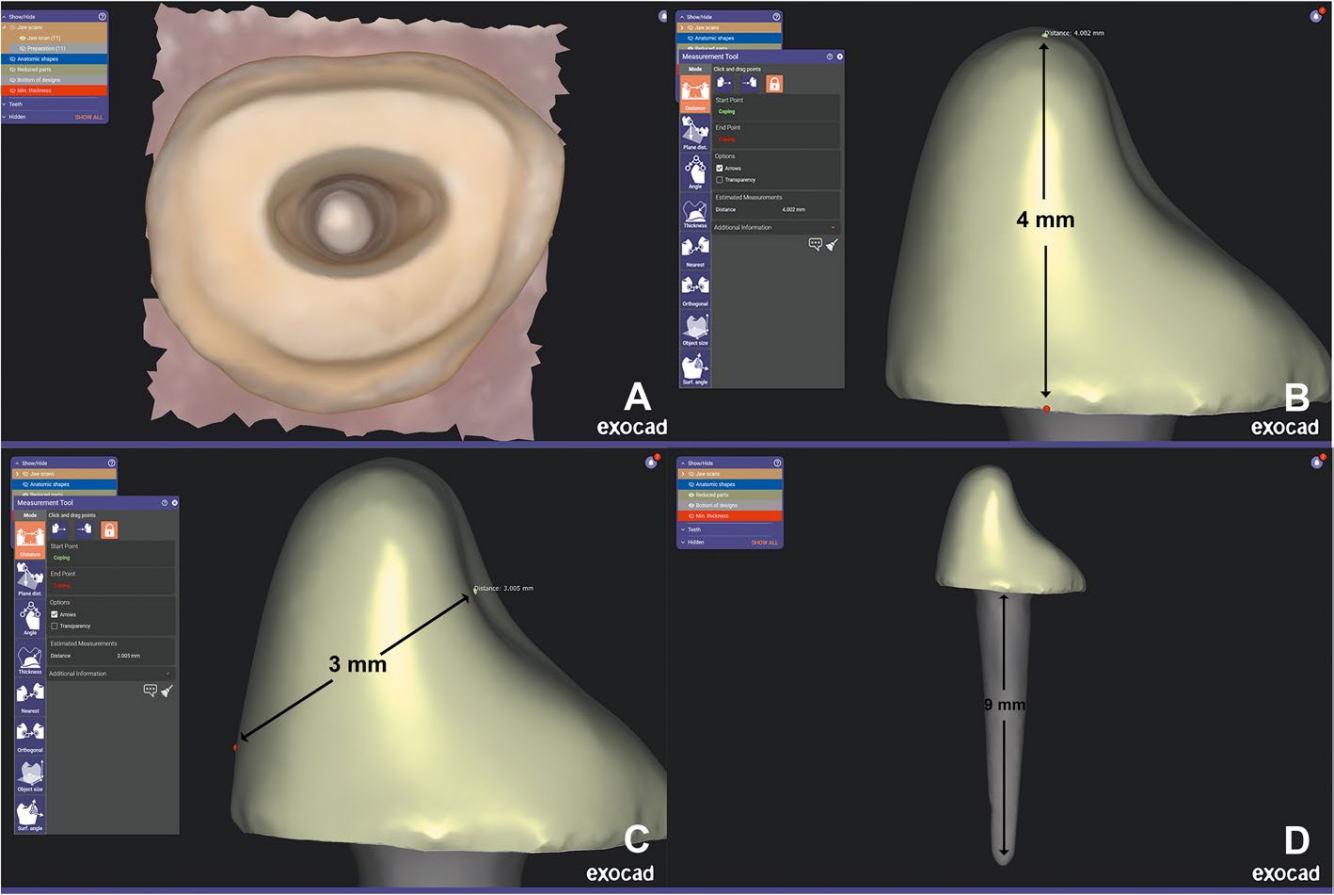
**A** Scanned specimens with post space. **B** and **C** CAD of post and core restoration showing core with 4 mm length and labiolingual diameter of 3mm measured diagonally. **D** Final CAD of post and core. CAD, computer-aided design

The post and core 3D models were designed using a dental CAD program (exocad Dental DB; exocad GmbH) with an 80 µm cement space for adhesive bonding [37]. The core had dimensions of 4 mm in height and 3 mm in thickness, measured diagonally from the fossa to the buccal surface, as illustrated in (Fig. 1), to simulate the abutment of prepared teeth. The CAD files of the post and core restorations were exported to a CAM program (HyperDent; FOLLOW ME! Technology) to be nested in the corresponding materials. A dental milling machine (CORiTEC 250i touch; imes-icore) was used for the fabrication of post and core restorations.

### Cementation of post and core restorations

The PEEK and FRC restorations were sandblasted using 50 µm aluminum oxide (Al2 O3) at 2 bar for 10 seconds [9, 29]. Following this, a silane coupling agent (SILAN-IT; iTena) was applied to the FRC restorations [29]. Visiolink primer (Visio.Link, bredent) was applied to the surface of PEEK posts, followed by a 90-second light-curing process [38, 39]. Polymer infiltrated ceramic network restorations were etched with 9% hydrofluoric acid (CERAM-ETCH; iTena) for 60 seconds, followed by rinsing with water for 60 seconds, air drying for 20 seconds, and the application of a silane coupling agent (SILAN-IT; iTena), with subsequent air drying in accordance with the manufacturer’s instructions [40].

To cement the post and core restorations in their respective teeth, a dual-cure resin cement (TOTALCEM; iTena) was utilized. The cement was placed within the post space of the teeth using a spiral rotary instrument (Lentulo; Dentsply Sirona) followed by the application of a static load of 2 kg for 5 minutes This ensured the full seating of the restorations and the creation of a uniform cement film thickness [9]. In this study, crown fabrication was not employed due to the acknowledged effect of crowns in augmenting fracture resistance values [39, 41–44]. The main aim of this research was to amplify the focus on the intrinsic behavior of the materials themselves.

### Aging of the specimens

The samples underwent 500 thermal cycles ranging from 5℃ to 55℃ with a dwell time of 1 minute and a 30-second transfer time using the thermocycling machine (Custom made in dental biomaterials dept; faculty of dentistry Alexandria University). Following the thermocycling process, all specimens were subjected to cyclic loading consisting of 50,000 load cycles at a frequency of 2Hz, with a load of 50N, utilizing a load cycling machine [45, 46]. Before testing, all specimens were stored in distilled water at 37°C for 7 days.

### Fracture resistance test

**Fig. 2:**
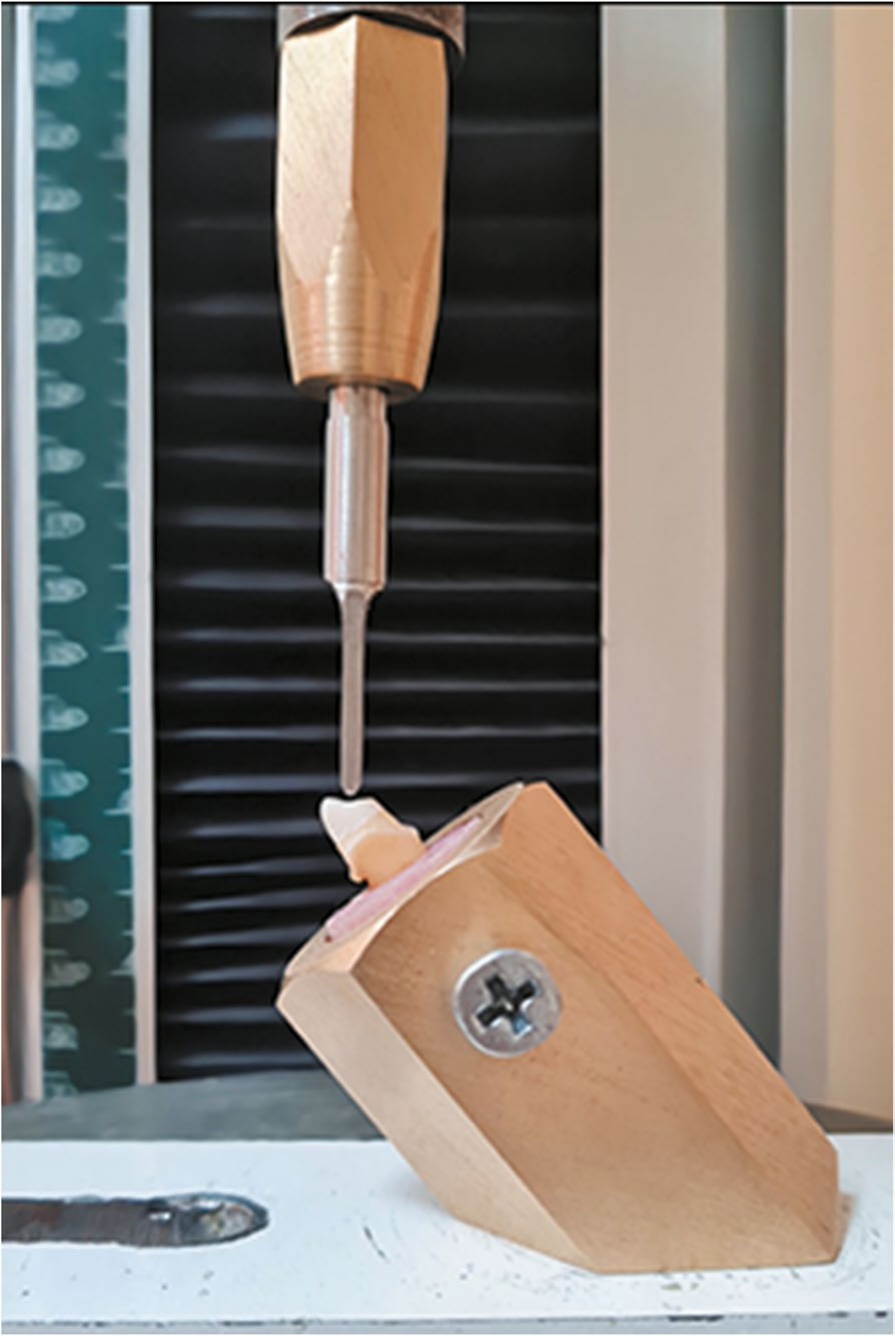
Compressive static load applied 45 $^\circ$ palatal to specimen

The specimens were mounted in a cylindrical metal holder with an inclination of 45-degree as shown in (Fig. 2). The specimens were subjected to fracture test using a universal testing machine (5ST; Tinius Olsen). A custom-made blunt rounded chisel head (2 mm in size) was used to apply force at a crosshead speed of 1 mm/min. The force was applied at a 135-degree angle to the tooth’s long axis, targeting the palatal surface, positioned 2 mm cervical to the incisal edge [47]. Each specimen’s failure load was recorded in Newton. Failure was defined as the point at which the strain-stress diagram abruptly dropped, and fracture resistance was recorded as the moment at which the loading force reached its maximum value [48].

### Assessment of mode of failure

Specimens, fragments and failure patterns were examined using stereomicroscope (Olympus; Olympus Co) at x4 magnification and scanning electron microscope (SEM) at x13 and x500 magnification with an accelerating voltage of 20 KV (Jeol JSM-IT200; Jeol Ltd).

In cases where failure manifested as a fracture in the core, post, or a tooth fracture occurring above the level of the cement-enamel junction, it was considered a favorable fracture that could potentially be repaired. Conversely, an unfavorable fracture encompassed root fractures occurring below the level of the cement-enamel junction, including vertical, horizontal, or oblique root fractures [49–51].

### Statistical analysis

Descriptive statistics were calculated as means standard deviation (SD), median, interquartile range (IQR), and range (Min – Max) for the fracture resistance variable, in addition to frequencies and percentages for the restorability variable. Normality was tested for the fracture resistance variable using descriptive statistics, plots (histogram and Q-Q plots), and normality tests. The variable showed normal distribution, so parametric analysis was adopted. Comparison between the three study groups was done using One-way ANOVA, followed by multiple pairwise comparisons using Bonferroni adjusted significance level. Comparison of restorablitiy was performed using Chi-square test. Significance level was set at *p* value <.05. Data were analyzed using IBM SPSS for Windows (Version 26.0)

## Results

The mean fracture resistance of all groups is shown in (Table 2). The highest mean fracture resistance was recorded in FRC (452.60 ±105.90 N), followed by PICN (426.76 ±77.99 N), and the lowest mean fracture resistance PEEK (286.16 ±67.09 N) are presented in (Table 2). The one-way ANOVA test indicated a statistically significant difference among the tested groups.

**Table 2:** Comparison of fracture resistance between the three study groups

	**Glass Fiber (*n*=13)**	**PEEK (*n*=13)**	**Vita Enamic (*n*=13)**
**Mean ±SD**	452.60 ±105.90^a^	286.16 ±67.09^b^	426.76 ±77.99^a^
**Median (IQR)**	447.00 (344.62, 558.76)	280.92 (219.95, 346.81)	400.17 (348.26, 506.29)
**Min – Max**	304.30 – 608.72	180.41 – 377.65	325.00 – 524.57
***F***	*F*= 15.54		
***P* value**	*P* value <.001*		
**Post-hoc comparisons**	Glass fiber compared to PEEK: .001*		
	Glass fiber compared to Vita Enamic: 1.00		
	PEEK compared to Vita Enamic: .003*		

*SD* Standard Deviation, *IQR* Interquartile Range, *Min* Minimum, *Max* Maximum, *F* One-way ANOVA was used

^a,b^Different letters denote statistically significant differences using Bonferroni adjusted significance level

^*^Statistically significant at *p* value <.05

Bonferroni adjusted post-hoc tests revealed that there was no statistically significant difference between FRC and PICN, while PEEK exhibited a statistically significant difference when compared to the other two groups (*P*<.05) (Table 2). There was no significant difference in failure mode and restorability between the tested groups (Table 3).

**Table 3:** Comparison of specimen restorability between the three study groups

	**Glass Fiber (*n*=13)**	**PEEK (*n*=13)**	**Vita Enamic (*n*=13)**
	***N* (%)**		
**Restorable**	5 (38.5%)	6 (46.2%)	7 (53.8%)
**Non-restorable**	8 (61.5%)	7 (53.8%)	6 (46.2%)
**Chi-square test**	X^2^: 0.62		
***P* value**	*P* value: 0.73		

**Fig. 3:**
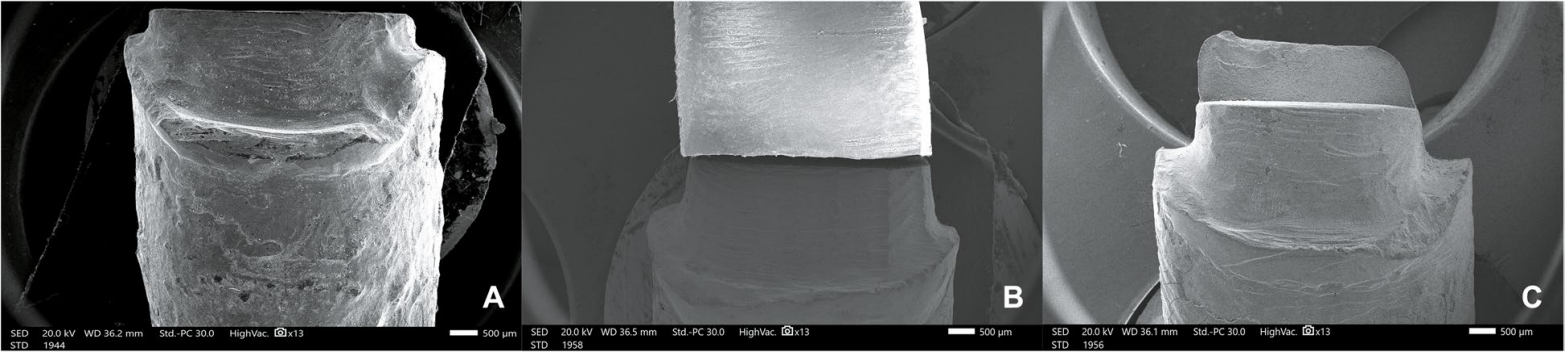
Scanning electron micrographs at 13 X magnification of three tested groups displaying favorable failure patterns: **A** FRC with core separated from post. **B** PEEK specimen with a gap between post and core restoration and tooth indicating dislodgement of restoration. **C** PICN specimen with fracture within core

**Fig. 4:**
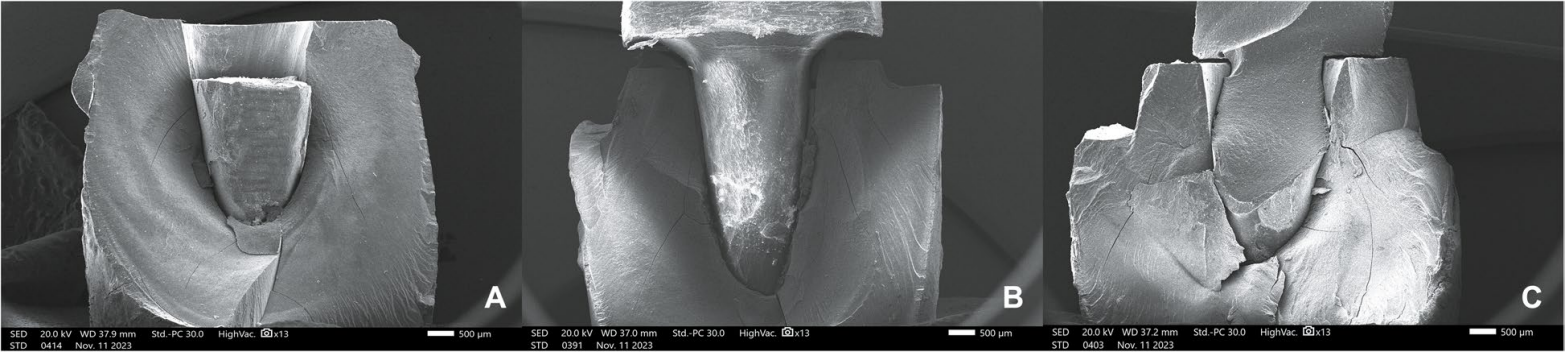
Scanning electron micrographs with 13 X magnification of three tested groups with non-favorable failure patterns. **A** FRC sample displayed fractures in both post and core restoration and vertical root fracture below CEJ. **B** PEEK sample exhibited vertical root fracture below CEJ, while post and core restoration remained intact. **C** PICN sample showed fractures in both post and core restoration, and vertical root fracture below CEJ

**Fig. 5:**
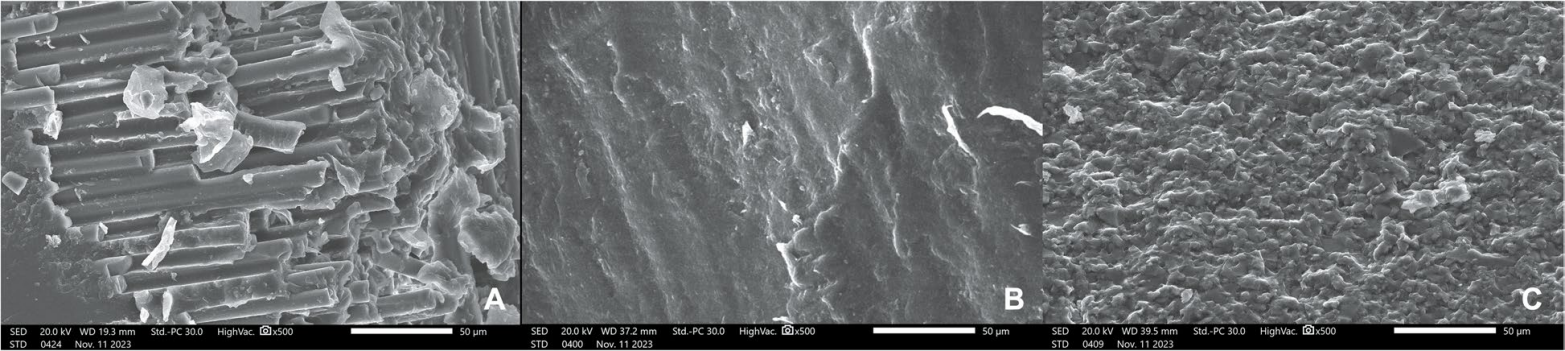
Scanning electron micrographs with 500 X magnification of fractured surfaces of three tested groups. **A** FRC specimen with failed post showing ruptured fibers with brush-like cracking. **B** PEEK specimen with intact external smooth surface. **C** Rough surface detected after fracture in PICN

**Fig. 6:**
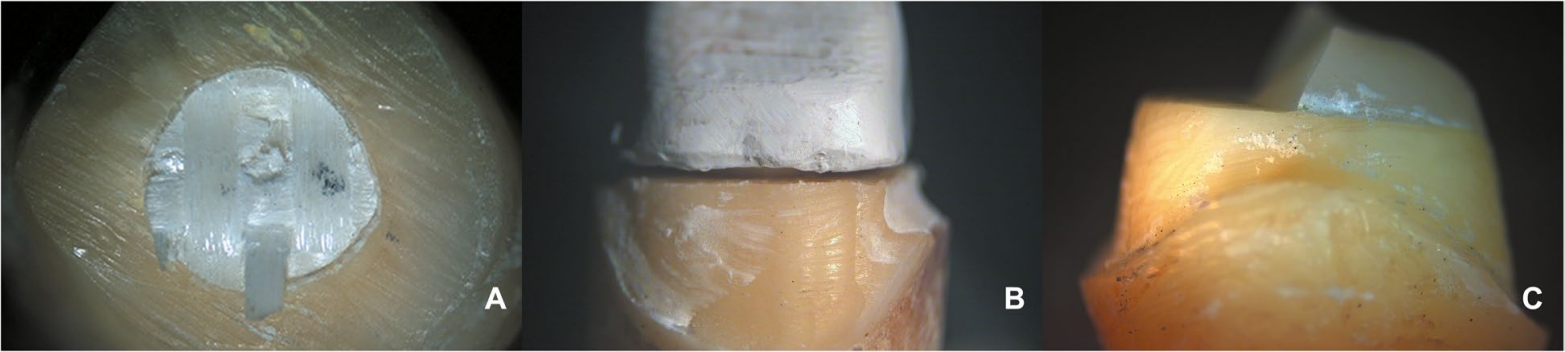
Stereomicroscope images with 4 X magnification showing favorable fracture patterns in three tested groups. **A** FRC sample displayed core separation. **B** PEEK sample exhibited post and core dislodgment. **C** PICN sample fractures confined within the core

**Fig. 7:**
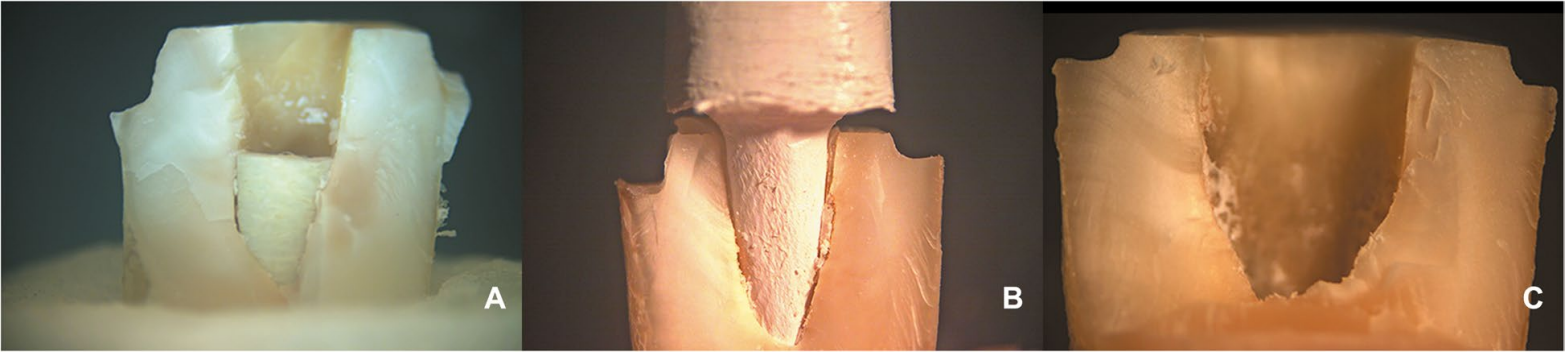
Stereomicroscope images with 4 X magnification showing nonfavorable fracture patterns in three tested groups: **A**, FRC sample displayed fractures in both post and core restoration and vertical root fracture below CEJ. **B**, PEEK sample exhibited vertical root fracture below CEJ, while post and core restoration remained intact. **C**, PICN sample showed fractures in both post and core restoration, and vertical root fracture below CEJ

Examining samples visually and using scanning electron microscope (SEM) and stereomicroscope imaging revealed distinct fracture patterns. In FRC samples, observations indicated favorable fractures marked by core separation that didn’t impact the post or tooth (Figs. 3A, 6A). Conversely, unfavorable outcomes included post damage and vertical root fractures below the CEJ (Figs. 4A, 7A). SEM images also revealed a pattern of brush-like cracking attributed to the rupture of these fibers (Fig. 5A )[52].

PEEK samples that exhibited favorable fractures underwent dislodgement without causing fractures in any component (Figs. 3B, 6B). Nevertheless, the unfavorable failures were distinguished by vertical root fractures occurring below the CEJ without any accompanying fractures in the post or the core (Figs. 4B, 7B). The SEM analysis notably showed a smooth surface on the PEEK post (Fig. 5B).

In PICN, the favorable failure mode involved fractures within the core segments (Figs. 3C, 6C), contrasting with the unfavorable pattern represented by a vertical root fracture below the CEJ and a fracture in the post (Figs. 4C, 7C). Additionally, SEM micrographs displayed a rough surface, showcasing the heterogeneity of components (Fig. 5C).

## Discussion

This in vitro study examined the fracture resistance of three aesthetic materials utilized as post and core restorations manufactured using CAD-CAM technology. Among the three tested groups, the custom PEEK post and core restorations showed the least values in fracture resistance. The fracture resistance of the custom FRC and PICN post and core restorations showed notably superior results when compared to the customized PEEK post and core restorations. The null hypothesis, suggesting no difference in fracture resistance among these three materials, was rejected.

In the present study, the selection of the maxillary central incisors was based on their pronounced need for aesthetic restoration. In addition to their characteristic feature of possessing a straight, single, and flared root canal. This choice was made to facilitate standardization and enhance validity of the study in comparison to utilizing artificial teeth. A standardized post space of 9 mm in length was created to align with the protocols followed in previous studies [43, 53].

An intraoral scanner was utilized to scan the teeth, including post spaces, enabling the fabrication of post and core restorations through a fully digital workflow. This method aimed to enhance precision, reduce time consumption, and confront the limitations associated with traditional methods. Alhasher et al. [8] proposed that employing intraoral scanners for constructing single-piece post and core restoration could offer a reliable alternative to conventional methods. Elter et al. [35] suggested that intraoral scanners are viable for capturing impressions of the post space when the post depth measures less than 14 mm, thus facilitating a streamlined impression procedure. Additionally, Vogler et al. [36] concluded that the fully digital chairside workflow, coupled with CAD-CAM-fabricated post and core, displayed superior accuracy of fit and practicality in impression-taking compared to conventionally fabricated cast post and core. Consequently, CAD-CAM technology holds the potential for enabling single-session restoration of endodontically treated teeth with customized post and core solutions, offering efficiency and precision in dental practice.

Previous studies employed ceramic materials with high elastic modulus such as lithium disilicate and zirconia to construct custom post and core restorations. It was found that there was stress concentration on root dentin resulting in catastrophic failure [44, 47, 54–58]. The investigated materials in the current study exhibit a modulus of elasticity close to dentin, approximately 18 GPa, [59] leading to a more uniform distribution of stress along the root. Consequently, this even distribution mitigates the occurrence of vertical root fractures.

In the present study, the exclusion of crown fabrication aimed to amplify the effect of post and core materials in the assessment of fracture resistance of endodontically treated teeth [41–44]. On the other hand, studies [40, 60, 61] that used a crown or coping to cover post and core restorations had fracture resistance higher than the current study, however, this might conceal the actual differences between the used custom made post and core materials. Thermal and mechanical aging have significant impacts on the bonding and fracture resistance of endodontically treated teeth restored with post and core restorations. Despite the relatively brief duration of thermomechanical aging in our study, the observed outcomes align with findings from comparable studies, [45, 46] which also reported similar statistical differences and failure modes. Notably, mechanical loading exerted direct effect on the integrity of the post and core restoration, while thermal cycling notably affected the interface of cemented surfaces, exacerbating the degradation of bond strength and mechanical properties of the post and core restoration [62, 63]. Moreover, several studies [9, 30, 42, 47, 55, 64] refrained from conducting artificial aging, emphasizing that their investigations were specifically focused on the materials and methodology rather than projecting outcomes under different conditions.

The applied force was oriented at a 135-degree angle in relation to the long axis of the tooth, targeting the palatal surface. This was achieved using a specially designed chisel head designed to emulate the lower central incisor, thereby replicating the clinical situation [47].

While PEEK exhibits comparable elastic modulus and flexural strength to dentin, excessive loading on PEEK post and core restorations tends to concentrate stress on the surrounding cement layer [19]. This often results in debonding, given that the sole bonding mechanism relies on mechanical interlocking attained through sandblasting [9]. Heightened stress can concentrate on the tooth, potentially causing catastrophic root fractures. The recorded values for PEEK posts and core restorations were 286.16 ± 67.09 N, aligning with findings by Özarslan et al. [65] who similarly assessed fracture resistance across PEEK, zirconia, and glass fiber. Their study revealed that the average failure loads of PEEK posts and core restorations were 306.7 ± 74.0 N.

Additionally, there is agreement with Teixeira et al. [20] who conducted a comparison of the fracture resistance between custom-made post-and-cores of PEEK and Nano-ceramic Composite. Their findings reported a mean fracture resistance of PEEK post and core restoration at 379.46 ± 119.8 N.

In contrast to our findings, Abdelmohsen et al. [39] investigated the fracture resistance of various post and core systems. They reported mean values of fracture resistance for CAD-CAM milled PEEK post and core restorations at 1055.25 ± 119.31 N. This discrepancy may be attributed to the study’s focus on premolars and the varied angulations of applied force.

The fracture resistance mean values for the PICN group in this investigation were measured at 426.76 ±77.99N. These findings were consistent with the observations of Spina et al. [43] where they reported mean fracture resistance values of 414.5 ±83.9N for PICN and 407.6 ±109N for FRC. Additionally, Elmaghraby et al. [45] also observed agreement in their research, noting fracture resistance mean values of 386.6 ±25.78N for one-piece post and core PICN restorations.

The findings in this study contrasted with those of Alkhatri et al. [30] who investigated the impact of materials on the apical extension of root fracture. Alkhatri et al. reported mean fracture resistance values of PICN as 271.06 ±69.57N. The variations in observed values could stem from employing diverse approaches in fabricating the post and core utilizing acrylic resin. These differences might arise due to distinct methodologies, such as variations in resin composition, application techniques, curing processes, or the specific protocols followed during fabrication.

The FRC group exhibited the highest mean failure load value of 452.60 ±105.90N. This finding is consistent with the results of Eid et al. [29] who investigated the fracture resistance and failure mode of endodontically treated teeth restored with custom-made FRC post and core restorations. They noted a mean fracture resistance of 367.06 ±72.34N for CAD-CAM glass fiber post and core restorations, further supporting the consistent performance of glass fiber reinforced composite materials in enhancing fracture resistance.

In the current study, the fracture resistance among the three tested groups fiber reinforced composite samples, PEEK, and polymer infiltrated ceramic network measured 452.60 ± 105.90 N, 286.16 ± 67.09 N, and 426.76 ± 77.99 N, respectively. These values exceed the recorded maximal occluding force generated by maxillary incisors, which stands at 146 ± 44 N [30]. This indicates that each of the three tested groups could be considered acceptable materials of choice for anterior tooth restoration requiring a customized post and core.

In terms of the failure mode, no significant difference was noted among the tested groups. PICN demonstrated the most favorable results, followed by PEEK, while the fewest number of restorable specimens were found in FRC, with counts of 7, 6, and 5, respectively. These findings align with the majority of previous studies [30, 43, 45, 61, 65].

Due to its relatively rigid molecular chain structure, PEEK demonstrates notable ductility, allowing for substantial deformation under unilateral stress during compression [20]. When subjected to stresses within its yield limit, the material undergoes elastic deformation. However, when these stresses surpass the yield limit, [19] PEEK experiences plastic deformation and bending without encountering fracture or chipping [21]. This behavior contributes to understanding the prevalent failure patterns observed in PEEK post and core restorations. Applied force tends to transfer to the intermediate cement, causing dislodgement without inducing fracture in any component, whether the post, core, or the tooth structure itself. Alternatively, this force concentration on the root can lead to a vertical root fracture as reported in the current study.

According to the study conducted by Özarslan et al. [65] which focused on comparing the fracture strength of endodontically treated teeth restored with different post-core systems, it was found that 40% of the specimens experienced decementation without fracture. This finding is consistent with our research results. On the other hand, Pourkhalili et al. [64] in their study on the fracture resistance of various post and core systems, reported that none of the specimens exhibited debonding. This disparity in results could be attributed to their use of premolars instead of anterior teeth and the application of force in a different direction in their study. Additionally, Kasem et al. [66] utilized customized PEEK post and core restorations for compromised teeth, documented a successful five-year follow-up. However, the favorable outcome observed can be explained by the ferrule effect of several millimeters used in the case report.

The failure of the FRC material stemmed from the rupture of its fibers. In instances where a fiber-reinforced material experiences failure, a crack initiates within the matrix and proceeds along the interface encircling a dispersed fiber, resulting in the rupture of the fiber itself. Subsequently, the load transfers to adjacent fibers, causing them to rupture in sequence. This mode of failure is commonly described as ‘brush-like’ cracking [37, 52]. The fractured samples in the PICN group exhibited a crack propagation failure mechanism, aligning with the observations made by Aboushelib et al. [24] They reported a diminished resistance in the polymeric matrix of resin-infiltrated ceramics, resulting in a linear crack and the formation of a slipstream path in the direction of crack propagation.

In the current study, the unfavorable outcomes were 61.5%, 53.8%, and 46.2% for glass fiber reinforced composite, PEEK, and polymer infiltrated ceramic network, respectively. However, these results still outperformed those reported by Hamdy et al. [55] where 80% of translucent zirconia post specimens were deemed non-restorable, as well as by Özarslan et al. [65] who found that 72.5% of zirconia post-core group samples were irreparable. Additionally, Alkhatri et al. [30] investigated the impact of post and core materials on the apical extension of root fracture in root canal treated teeth. It was observed that in the PICN group, the extension of root fracture tended to be more coronal compared to the metal and zirconia groups, likely due to variations in the modulus of elasticity among the tested materials. Additionally, Bittner et al. [57] conducted a study comparing a one-piece milled zirconia post and core with other post-and-core systems. Their findings indicated that all specimens in the one-piece milled zirconia post and core group experienced catastrophic root failure.

Similar to any other in vitro study, it’s important to note that the current research couldn’t entirely replicate in vivo conditions as most fractures occurring in vivo would likely be due to fatigue conditions rather than a compressive static load. This study doesn’t fully replicate the clinical situation as it doesn’t consider the use of crown restoration, which, when applied with adequate ferrule, significantly increases the fracture resistance of endodontically treated teeth (ETT).

## Conclusions

Within the limitation of this study, it could be concluded that:

- 1. CAD-CAM milled FRC and PICN post and core restorations possess satisfactory fracture resistance in comparison to PEEK post and core restorations.
- 2. In terms of failure mode, there were no noteworthy distinctions observed among the tested groups regarding the restorability of teeth after failure.

## Data Availability

The datasets generated and analysed during the current study are available from the corresponding author on reasonable request.

## Abbreviations

EET: Endodontically treated teeth
CAD-CAM: Computer aided design and computer aided manufacturing
FRC: Fiber reinforced composite
PEEK: Polyether ether ketone
PICN: Polymer infiltrated ceramic network
